# Structure of a translocation signal domain mediating conjugative transfer by type IV secretion systems

**DOI:** 10.1111/mmi.12275

**Published:** 2013-06-14

**Authors:** Adam Redzej, Aravindan Ilangovan, Silvia Lang, Christian J Gruber, Maya Topf, Klaus Zangger, Ellen L Zechner, Gabriel Waksman

**Affiliations:** 1 Institute of Structural and Molecular Biology, UCL and BirkbeckMalet Street, London, WC1E 7HX, UK; 2 University of Graz, Institute of Molecular BiosciencesHumboldtstrasse 50, 8010, Graz, Austria; 3 University of Graz, Institute of ChemistryHeinrichstrasse 28, 8010, Graz, Austria

## Abstract

Relaxases are proteins responsible for the transfer of plasmid and chromosomal DNA from one bacterium to another during conjugation. They covalently react with a specific phosphodiester bond within DNA origin of transfer sequences, forming a nucleo-protein complex which is subsequently recruited for transport by a plasmid-encoded type IV secretion system. In previous work we identified the targeting translocation signals presented by the conjugative relaxase TraI of plasmid R1. Here we report the structure of TraI translocation signal TSA. In contrast to known translocation signals we show that TSA is an independent folding unit and thus forms a *bona fide* structural domain. This domain can be further divided into three subdomains with striking structural homology with helicase subdomains of the SF1B family. We also show that TSA is part of a larger vestigial helicase domain which has lost its helicase activity but not its single-stranded DNA binding capability. Finally, we further delineate the binding site responsible for translocation activity of TSA by targeting single residues for mutations. Overall, this study provides the first evidence that translocation signals can be part of larger structural scaffolds, overlapping with translocation-independent activities.

## Introduction

Type IV secretion systems (T4SS) are protein complexes spanning bacterial cell membranes (Alvarez-Martinez and Christie, 2009; Wallden *et al*., 2010; Zechner *et al*., 2012). They are used to transport biomolecules such as proteins, nucleic acids and nucleoprotein complexes across the cell envelope. They can be divided into three subclasses: (i) the effector protein translocation T4SS which are responsible for the delivery of effector proteins into the cytoplasm of eukaryotic cells (Terradot and Waksman, 2011), (ii) the conjugative systems which transfer DNA or nucleoprotein complexes from a donor to a recipient strain in a cell contact-dependent manner (Fronzes *et al*., 2009), and finally (iii) T4SS which release DNA into or mediate uptake from the extracellular milieu (Hamilton *et al*., 2005). T4SS have broad clinical significance not only as virulence factors but also as a major vehicle for the dissemination of antibiotic resistance genes in clinical and natural environments.

T4SS in Gram-negative bacteria are typically composed of 12 proteins termed VirB1-11 and VirD4 based on the prototypical *Agrobacterium tumefaciens* T-DNA delivery system. The VirB proteins form a large macromolecular translocation channel embedded in both the inner and outer membranes (Wallden *et al*., 2010). T4SS substrates are thought to be recruited to the VirB machinery by the VirD4 ATPase, a protein which couples substrate recruitment to secretion and therefore commonly referred to as the type IV coupling protein (T4CP) (Cabezón *et al*., 1997; Schröder and Lanka, 2003; Alvarez-Martinez and Christie, 2009). T4CPs mediate multiple protein–protein interactions with cytoplasmic and inner membrane components of the secretion system. ATPase activity is associated with the release and unfolding of complexes between substrates and specific chaperones and is required to energize the secretion process.

Conjugation systems are the largest and most widely distributed of the T4SS subtypes. The general mechanism of nucleoprotein transfer by these systems is well characterized (de la Cruz *et al*., 2010). Multiple proteins assemble on the plasmid origin of transfer (*oriT*) to form the relaxosome. This complex prepares the single strand of plasmid DNA destined for transfer (T-strand) via the nicking–closing activity of the relaxase enzyme. Initiation of transfer requires cleavage at a specific position *nic*, within *oriT*. The reaction is mediated by a tyrosine residue of the enzyme so that a covalent protein–DNA adduct is formed. This nucleoprotein complex is specifically recognized as a substrate by the T4CP and actively pumped through the transport apparatus. Once in the recipient the relaxase-T strand intermediate restores the original circular plasmid molecule by reversion of the strand transfer reaction.

**Figure 1:**
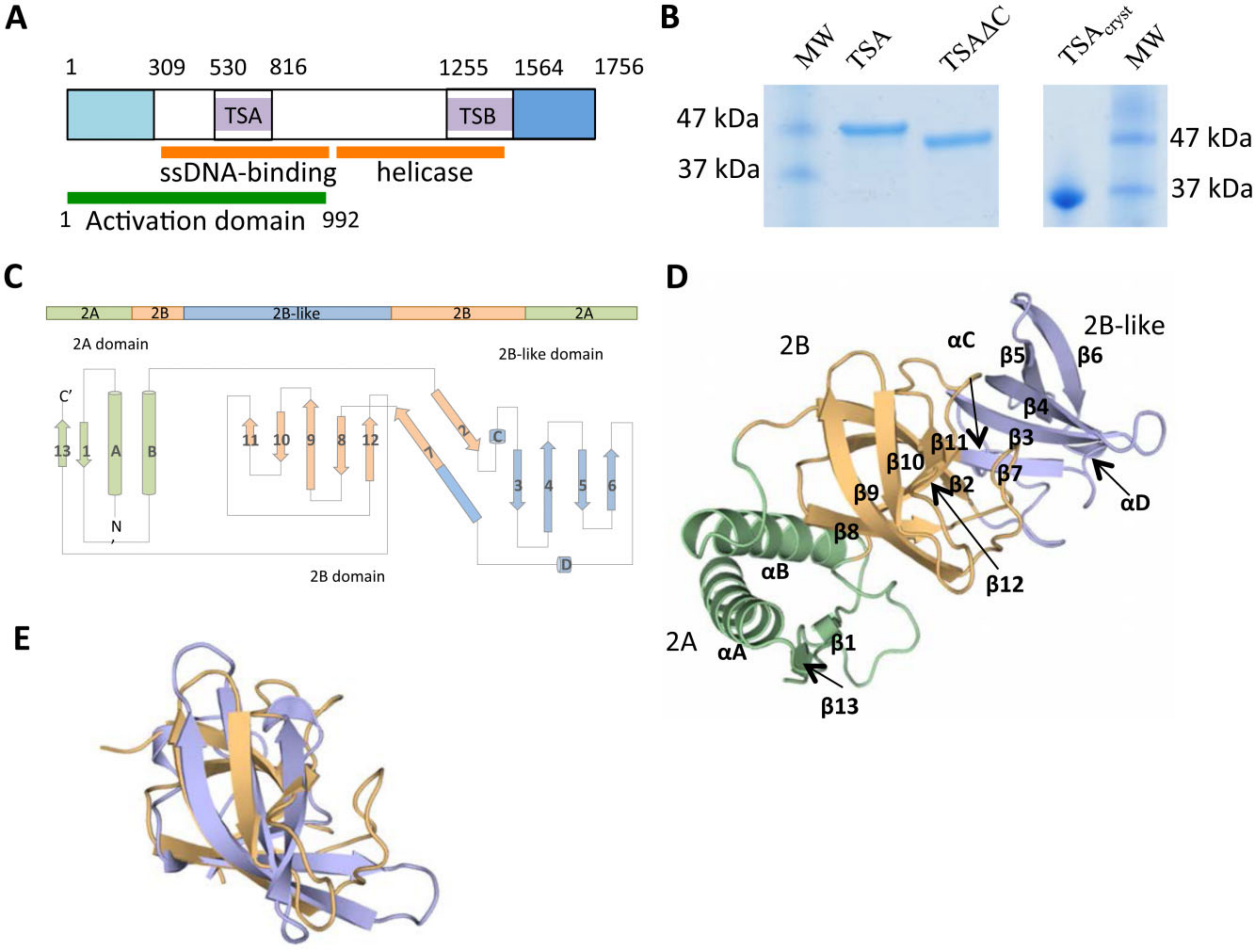
Crystal structure of TSA.A. Schematic diagram of the domain structure of TraI. The N-terminal relaxase (light blue), the two translocation signal domains (TSA and TSB), and the C-terminal domain (dark blue) are indicated, as well as the ssDNA and helicase domains (orange). The domain involved in T4SS channel activation via TraD-relaxosome interaction in shown in dark green. Boundary residues for each domain except the RecD domains (the boundaries of which are still unclear) are reported above the diagram.B. Purification of TSA. Two bands were observed (left panel), one full-length (TSA) and the other resulting from C-terminal degradation (TSAΔC). Only TSAΔC crystallized. During crystallization, TSAΔC underwent further proteolytic degradation at the N-terminus, resulting in a band shown at right (TSAcryst).C. Topology diagram of TSA. TSA contains three domains (here represented in green, orange and blue) which, due to their structural similarity with domains 2A and 2B of SF1 helicases, are termed ‘2A’, ‘2B’ and ‘2B-like’ (see Fig. 2). This diagram illustrates the overall organization of the sequence and domain structure as well as secondary structure composition.D. Crystal structure of TSA. The three structural domains are in ribbon representation and are colour-coded as in (C).E. Structural homology between the 2B and 2B-like domain. The middle and C-terminal domains of TSA were superimposed. The two domains are in ribbon representation in the same orientation as domain 2B in (D).

In the paradigm F and related R1 plasmid systems the 192 kDA protein TraI is the substrate of the T4SS encoded by the *tra* genes (Everett and Willetts, 1980; Reygers *et al*., 1991; Lang *et al*., 2010; Dostal *et al*., 2011). This bifunctional protein includes both the relaxase activity and a helicase that assists unwinding of the T-strand. The relaxosome contains the plasmid encoded proteins TraM and TraY, and the host genome-encoded protein IHF. All of these factors assist relaxase in nic-cleavage and TraM and IHF additionally stimulate the helicase (Nelson *et al*., 1995; Kupelwieser *et al*., 1998; Csitkovits and Zechner, 2003; Sut *et al*., 2009). Although an additional role for these accessory proteins is not entirely clear, TraM is thought to recruit the relaxosome to the T4SS via interaction with the T4CP TraD (Lu *et al*., 2008; Wong *et al*., 2011; 2012). TraI contains four domains (Fig. 1A): a *c.* 300 residue relaxase domain, followed by a single-stranded DNA-binding domain (extending to residue 822) and a helicase domain (the boundaries of this domain are still undefined but the domain spans approximately a region encompassing residues 980–1500), and a *c.* 200 residues domain at the C-terminus (Dostal and Schildbach, 2010; Lang *et al*., 2011). Co-ordinated regulation of ssDNA binding between the relaxase domain, the ssDNA-binding domain, and the helicase domain is thought to provide a major control point for transfer initiation; once cleaved and covalently attached to the relaxase domain, the plasmid DNA is transferred to the helicase-associated site to activate ATP-dependent unwinding. Interaction between TraI and the T4CP TraD is also key to controlling initiation: TraD indeed stimulates the relaxase and helicase activities (Mihajlovic *et al*., 2009; Sut *et al*., 2009). Moreover evidence is emerging that a complex of TraI bound simultaneously to *nic* and to the T4CP has a key function in activating the opening of the T4SS channel (Lang *et al*., 2011). To study this mechanism, Lang *et al*. used group 1 RNA phage which utilize conjugative pili as receptors. The phage genome of ssRNA with a protein attached to the 3′ end gain access to the cell interior by a process not yet defined but requiring the channel subunits and the T4CP. Docking of a relaxosome substrate to the transport apparatus is necessary for phage nucleoprotein penetration of the host cell. A minimal functional domain termed ‘the activation domain’ of TraI comprising the N-terminal 992 residues including a catalytically active relaxase is sufficient to enable nucleoprotein uptake (Fig. 1A; Lang *et al*., 2011).

**Figure 2:**
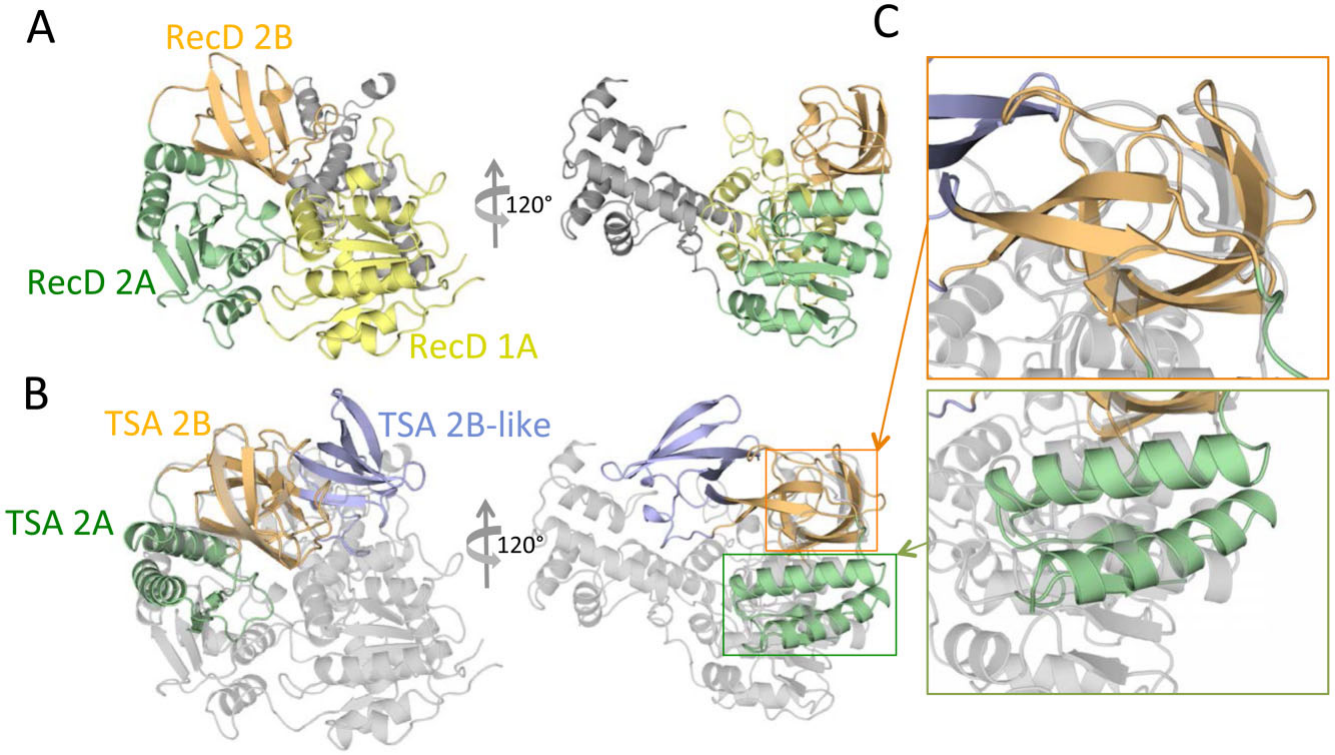
Structural homology between TSA and RecD helicase.A. Domain structure of RecD. The structure of a RecD homologue (RecD2 from *Deinococcus radiodurans*; PDB ID PD 1274) is shown in ribbon representation and in two orientations, 120° apart along the vertical axis. Domains 1A, 2A and 2B of RecD2 are colour-coded yellow, green and orange respectively.B. Superposition of the structure of TSA onto the structure of RecD2. RecD2 is in the same orientation as in (A) but is colour-coded in grey. TSA is in the same orientation and same colour-coding as in Fig. 1D. Orange and green rectangles indicate the regions, the details of which are shown in (C).C. Zoom-in on the superimposed parts. The regions within the orange and green rectangles in (B) are zoomed in to provide a visual illustration of the structural superposition between equivalent domains in TSA and RecD2. As can be seen, the 2A and 2B domains of TSA superimpose well onto parts of the 2A and 2B domains of RecD2. However, the 2B-like domain is shown not to be a part of the helicase domain.

Two different mechanisms for the recruitment of plasmid-loaded relaxosomes to T4CPs have been proposed. In F-like systems TraM binds to *oriT* and has been described to bind to the C-terminal domain of TraI (Ragonese *et al*., 2007); thus, by mediating binding to TraD, TraI and the DNA, TraM might mediate recruitment of the relaxosome to the coupling protein. As an alternative mechanism, direct binding of relaxases to T4CPs has been reported in some systems (Szpirer *et al*., 2000; Schröder *et al*., 2002; Cascales and Christie, 2004), but this has not been observed with R1 proteins (A. Redzej, unpublished).

The features of a substrate protein that specify it for secretion have been analysed in a few systems. In some cases short, C-terminal, signal sequences of positively charged or hydrophobic residues serve as export signals for the proteins that contain them (Nagai *et al*., 2005; Vergunst *et al*., 2005). A second group, which includes TraI, have larger, internally positioned signals. These regions of TraI, called translocation signals (TS) A (530–816) and B (1255–1564), target the relaxosome to the secretion machinery (Lang *et al*., 2010). Although each TS alone can confer substrate transport specificity these regions share nearly no primary sequence similarity except for a small consensus motif similarly present in the TS of the R1162 relaxase MobA (Parker and Meyer, 2007). TraI TSs are widely conserved within the MOB_F_ and MOB_Q_ families of relaxases (Lang *et al*., 2010).

In this study we report the crystal structure of the TSA domain of the TraI protein from the R1 plasmid. We show that TSA is composed of three domains, each displaying structural homologies with SF1B helicase family domains. Based on the TSA structure, site-directed mutagenesis was performed and the influence of the mutations on relaxase translocation by the T4S system was evaluated, thereby identifying the putative recognition interface within TSA that is involved in specific substrate recognition and recruitment to the T4S machinery.

## Results

The DNA encoding the TSA (530–816) region of TraI of the R1 plasmid system (Fig. 1A) was cloned in an expression vector downstream of a sequence encoding a His_6_-tag and an Enterokinase cleavage site. The resulting N-terminally tagged protein was expressed in *Escherichia coli*. After Ni-affinity chromatography, two fragments were observed on SDS-PAGE, one corresponding to TSA's expected size (33.7 kDa) and the other shorter at the C-terminus by *c.* 2–3 kDa, TSAΔC [this shorter fragment still contained its N-terminal His_6_-tag as demonstrated using Western blot analysis and anti-His antibodies (results not shown)]. These fragments could be separated by hydrophobic interaction chromatography (Fig. 1B). Both fragments were subjected to crystallization trials; however, only the smaller fragment crystallized. Analysis of the protein crystals on an SDS-PAGE indicated that, in fact, an even smaller fragment had crystallized, TSA_cryst_: N-terminal sequencing of the crystallized fragment showed the fragment to start at residue 570 (Fig. 1B). We have shown that removal of residues 530–568 lowers only moderately the relative efficiency of translocation, indicating that the crystallized fragment maintains functional integrity (Lang *et al*., 2010). No attempt was made to characterize the C-terminal sequence of the crystallized fragment. This fragment of TSA crystallized in the space group P41212 with one molecule in the asymmetric unit. The structure was solved using the single wavelength anomalous dispersion phasing method (Rice *et al*., 2000) from a SelenoMethionine (SeMet)-substituted variant of TSA (TSA contains six methionines), and refined to a final resolution of 1.85 Å (Table S1).

A model was built between residues 575 and 786. No electron density was observed for sequences on either side of these boundaries, indicating structural disorder beyond these sequences. The crystal structure of the TSA domain of the R1 plasmid is composed of three structural domains named ‘2A, 2B and 2B-like’ for reasons that are explained below. The 2A domain contains both the N-terminal and C-terminal ends of the TSA_cryst_ and is formed by two α-helices and two β strands (Fig. 1C and D, green). A second and third domains, 2B and 2B-like, are structurally similar (Fig. 1E) and their fold belongs to the SH3-like fold family (orange and blue in Fig. 1C and D respectively). The 2B domain is composed of six β strands (β2, β8, β9, β10, β11, β12) in an anti-parallel arrangement forming a central hydrophobic core. The 2B-like domain is composed of four β strands (β3, β4, β5, β6), two short helices (C and D) and shares a strand (β7) with the 2B domain (Fig. 1C). Domain 2B is made of sequences inserted within domain 2A, while domain 2B-like is made of sequences inserted within domain 2B (Fig. 1C).

A structural homology search using the DALI server (Holm and Rosenstrom, 2010) was performed which resulted in the identification of domain 2 of SF1B helicases as TSA's closest structural homologue: the RecD subunit of the RecBCD helicase ranked first with a Z-score of 8.9 (a highly significant score), followed by other SF1B helicase structures. SF1B helicase structures are typically composed of two domains, 1 and 2 (Subramanya *et al*., 1996; Korolev *et al*., 1997), each often split into two, subdomains A and B, with subdomain B composed of a sequence inserted in the middle of the sequence of subdomain A. This domain structure is illustrated in Fig. 2A using the structure of RecD2 from *Deinococcus radiodurans* as a typical example of a helicase structure. In this figure, three of the four subdomains, 1A, 2A and 2B, are colour-coded in yellow, green and orange respectively; domain 1B is not coloured as it is very small in RecD2. The structural superposition of TSA and RecD2 resulting from the DALI search is shown in Fig. 2B. As can be seen, two domains of TSA superimpose well with parts of subdomains 2A and 2B of RecD2 and, accordingly, are named TSA 2A and 2B domains (in green and orange in Figs 1D and 2B; details in Fig. 2C). The root-means-square deviation (rmsd) in Cα atoms between TSA and RecD2 equivalent domains 2A and 2B is 2.7 Å. The third TSA domain (in blue in Fig. 1D) superimposes well with the 2B domain of TSA (rmsd of 2.4 Å) and thus is named ‘2B-like’ (Fig. 1E). Thus, due to the high structural homology between TSA and SF1B helicase subdomains, we can conclude that TSA is part of a helicase structure, its two first domains, 2A and 2B matching parts of the structure of the 2A and 2B domains of SF1B helicases. Its third domain, 2B-like, is not part of the helicase fold but instead protrudes out as a separate domain which, in the TSA-RecD2 superposition presented in Fig. 2B, does not clash with any of the other domains of RecD2. Thus the 2B-like domain of TraI constitutes a previously uncharacterized addition to the helicase fold, which here serves as a signal recognition surface linked to substrate transport.

We next attempted to identify which surfaces in TSA likely mediate substrate translocation.

**Figure 3:**
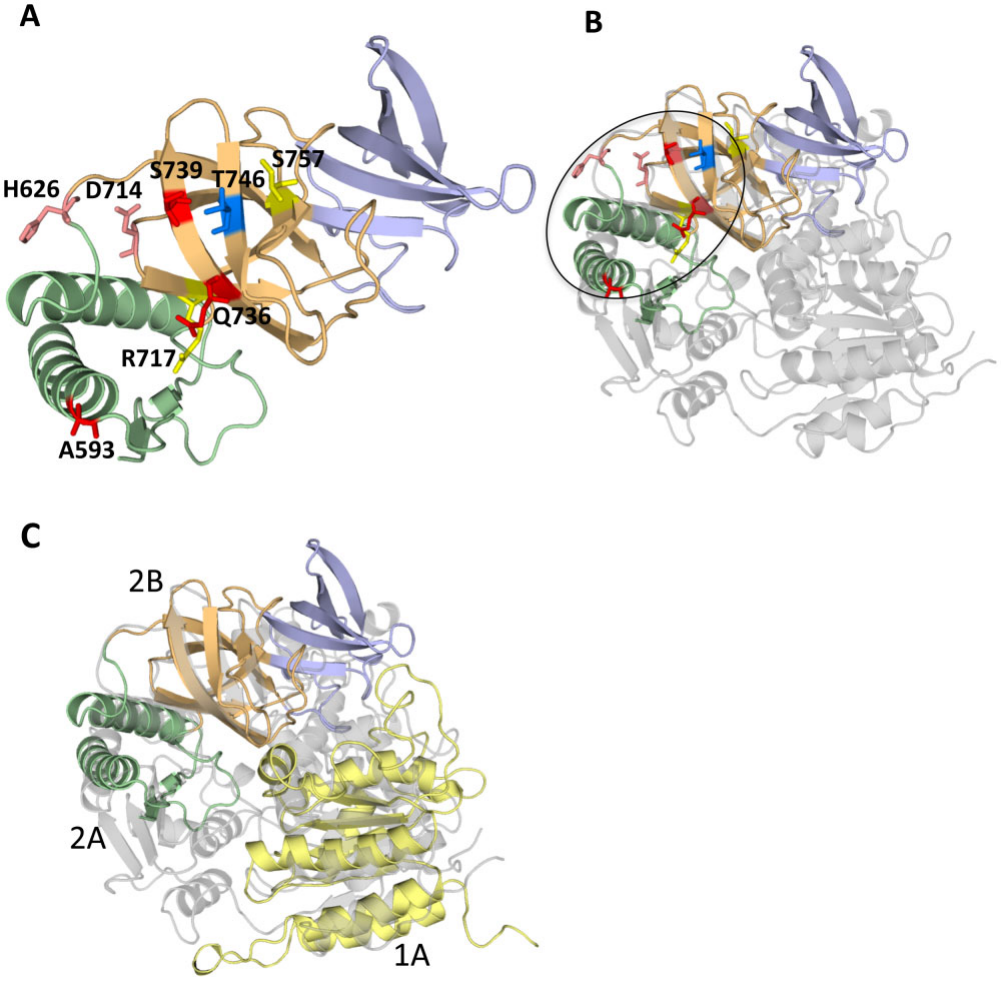
Mapping of investigated surface residues onto the structure of TSA.A. Mapping of the surface residues mutated in this study. Residues are shown in stick representation. Colour-coding of residues is as follows: red, pink, yellow and blue depending on whether mutations at these locations impairs TSA translocation function significantly (red), mildly (pink), not at all (yellow) or stimulate TSA translocation function (blue).B. Structural alignment of TSA and RecD2 showing the location of the surface residues in the context of the full-length helicase domain.C. Superposition of the structures of TSA (colour-coded as in the other panels) and of the 381–569 structure (Wright *et al*., 2012; in yellow) onto the RecD2 structure (in grey). Three domains of TraI ssDNA-binding domain have homologies with three domains of the helicase fold (1A, 2A and 2B), suggesting that the ssDNA-binding domain of TraI is a vestigial helicase that has retained ssDNA-binding activity and has also evolved to function as a TS.

The TSA region of TraI from R1 plasmid differs from that of the F plasmid by only two residues, S757 and H626 in R1, substituted for T757 and L626 in F. Despite this minor variation, the T4SS expressed by these two closely related plasmids can discriminate between the two TraI TSA sequences and selectively translocate only the cognate fragment. Exchanging residue 626 of R1 TraI for its counterpart in F was shown to change the substrate selection fidelity from R1 to F for TSA translocation (Lang *et al*., 2010). The crystal structure shows that these residues are located within the 2B domain and on the concave side of the arch-like TSA structure (Fig. 3A). No plasmid-specific discrimination was observed for TSA translocation due to residue 757 (Lang *et al*., 2010), so this position may lay outside the crucial recognition region. To define this region in more detail we carried out Ala and Asn scans on residues T746, Q736, S739 and D714 (see location of these residues in Fig. 3A). We also targeted residue 717 and 593, with the following rationale in mind: R717 is conserved in TSA, TSB, and also MobA of plasmid R1162 and is part of a small consensus motif; substitution of R717 by Gln in TSB was shown to affect substrate translocation (Lang *et al*., 2010); a nearby insertion (LDR) in MobA also blocked translocation (Parker and Meyer, 2007), thus, we mutated R717 to Gln in TSA. Concerning residue 593 (an Ala in R1 and F TraI proteins), insertion of a 31 residues sequence after this residue in the TraI relaxase encoded by the F plasmid eliminated detectable translocation of TSA (Haft *et al*., 2006; Lang *et al*., 2010); so we also sought to evaluate the effect of mutating this residue to a more bulky Val side-chain. Note that both R717 and A593 are also in the 626/757 region (see oval in Fig. 3B).

**Figure 4:**
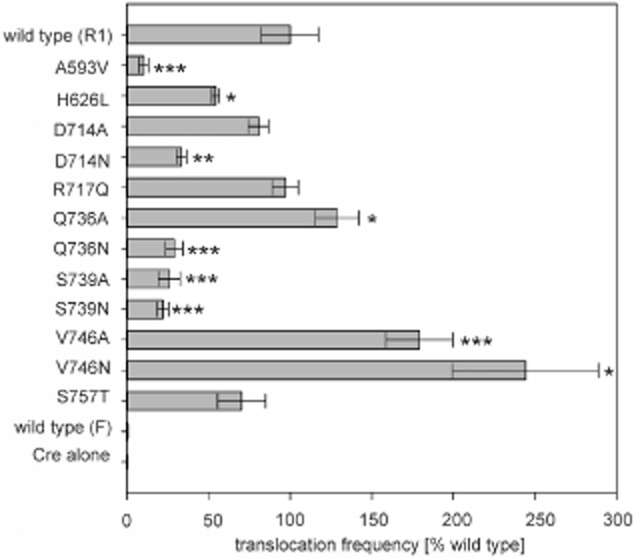
Translocation assay of TSA variants. Relative translocation frequencies (recombinants per donor cell) of the indicated variants (left) were compared with wild-type Cre-TraI_1–992_ from plasmid R1 (grey bars). Values (expressed as percentage of wild type) represent the mean of at least three independent experiments. Standard deviations are shown (*P < 0.05; **P < 0.01; ***P < 0.001).

The different site-specific mutants were generated and tested in the CRAFT assay, which is based on the fusion of a Cre reporter enzyme to the protein of interest. Secretion of the fusion protein is monitored via Cre-mediated recombination of a reporter cassette in recipient cells. Even though the TSA region is the minimal fragment sufficient for protein translocation to the recipient strain during conjugation (Lang *et al*., 2010; translocation frequency ∼ 2 × 10^−5^), site-specific mutations were introduced into the TraI_1–992_ construct (translocation frequency ∼ 1 × 10^−4^) to improve the readout of the assay and to enable comparisons with another functional test requiring productive TraI binding interactions as described below. The expression and stability of all the mutant variants fused to Cre were evaluated by Western blot analysis (Fig. S1) as well as by measuring the recombination efficiency upon transformation (Fig. S2). Recombination efficiency was determined by introducing the same amount of DNA from each genetic construct investigated into the reporter strain, and calculating the number of chloramphenicol-resistant colonies (recombinants) divided by the number of ampicillin-resistant colonies (transformants). The results indicated that Cre-TraI_1–992_ could catalyse recombination with ∼ 80% the efficiency of Cre alone, and the rest of the fusion proteins showed recombination rates between ∼ 80% and ∼ 120% of wild type (Fig. S2). The CRAfT assay results are presented in Fig. 4 and mapped onto the structure of TSA in Fig. 3.

Five mutations resulted in significant loss of translocation activity: A593V, D714N, Q736N, S739A and S739N, indicating that four residues, 593, 714, 736 and 739 play an important role as recognition surfaces for substrate export. In addition, mutation of residue 746 to Ala or Asn resulted in significant increase in substrate transport, also suggesting a role for this residue in translocation. H626 also appears to be involved since mutation to its equivalent residue in F TraI does reduce translocation efficiency, albeit to a lesser extent than the residues mentioned previously. Finally, mutation R717 to Asn or 757 to Thr has no effect. Thus, these results clearly define a surface on TSA that is responsible for substrate recognition and transport (shown in the oval in Fig. 3B). Since direct binding between TraI and TraD has never been observed (indicating either that the interaction is dominated by a rapid k_off_ or that a third partner might be involved either by providing additional binding energy or by inducing conformational changes in one of the binding partners resulting in additional surfaces being involved in binding), we could not confirm whether any of these mutations either reduces or enhances interactions between the two proteins.

It was previously observed that infection of cells harbouring the R1 plasmid by the R17 phage can be achieved in the presence of a minimal fragment TraI_1–992_ comprising the relaxase catalytic activity, the relaxase-associated ssDNA binding site and TSA. Indeed, this protein fragment together with the DNA is sufficient to activate the T4SS channel for phage entry (Lang *et al*., 2011). This raises the question as to whether the proposed recognition interface on TSA is necessary for R17 phage infection and in turn for the activation of the T4SS channel. In order to test this hypothesis all the mutants that have been shown to participate in substrate recognition have been tested in a phage assay. Interestingly, all of them were able to support phage infection at the same level as the wild-type protein (Fig. S3). This indicates that although mutation of these residues in the TraI_1–992_ impair efficient translocation of the protein fragment, they were not sufficient to impair the function of the TraI activation domain in nucleoprotein import. This finding will need to be further investigated but indicates that a wider range of protein–protein and/or protein–DNA interactions are involved in the activation process.

## Discussion

Relaxases are important proteins involved in DNA transport by T4SS. These proteins form covalent nucleo-protein complexes called relaxosomes which serve as substrate for T4SS-mediated transport. Previous studies have shown that two regions of the TraI relaxase encoded by the R1 plasmid act as recognition signals, termed TSA and TSB. This is unusual: typically, transport signal sequences are short (5–25 residues) sequences located at either the N- or C-terminal end of translocated substrates. For example, translocation signals for the *A. tumefaciens* T4S system or for the *Dot/Icm* T4S system from *Legionella pneumophila* are small clusters of positively charged or hydrophobic residues located at the C-terminus of the translocated substrates (Nagai *et al*., 2005; Vergunst *et al*., 2005). Translocation signals can however be more complex: for example, the *Bartonella* effector proteins have bipartite translocation signals where a charged C-terminal residues region is combined with one or more copies of a second motif located within the so-called ‘Bep intracellular delivery (BID) domain’ inside the protein sequence (Schulein *et al*., 2005). In TraI, TSA and TSB are much longer sequences and they are located within the protein sequence, not at the ends. Both function independently from each other.

Because signal sequences are usually short, of unusual amino acid composition, and generally located at either the N- or C-terminal ends of translocated substrates, they are believed to be unstructured. Moreover, attempts at structurally characterizing internal translocation signal regions of secreted effectors have been inconclusive. For example, Voulhoux *et al*. (2000) identified a domain within exotoxin A, a type II secretion system effector, which appears to act as a translocation signal; however, the functional characterization of this domain was based on removing entire secondary structures (the domain contains six helices) and the only helix (αF) deletion that resulted in significantly reduced translocation is a crucial structural secondary structure, the removal of which was bound to drastically perturb the entire structure. A second example is HasA, a type I secretion system effector (Masi and Wandersman, 2010), where residues within the HasA sequence were shown to affect translocation of the substrate but did not cluster within any particular region of the structure, indicating that the effects on translocation were likely conformational, rather than impairing the targeting of HasA to the secretion system.

Thus, with the work presented here, we demonstrate for the first time that translocation signal sequences can (i) fold into defined, well-characterized structures, and (ii) be integrated structurally within the more extended scaffold of proteins of well-known functions.

With its two terminal ends located near one another and interacting in a two-stranded beta-sheet, TSA indeed forms an independent folding unit, i.e. a self-contained structural domain. Its three subdomains, 2A, 2B and 2B-like, are formed by sequence inserted within one another, 2B within 2A and 2B-like within 2B. There is no precedent for such an observation. Yet, folded TSs might be more widespread that originally believed: for example, the BID region of Bep proteins might also form folded domains, although it cannot be excluded that the actual sequences responsible for translocation by BID regions might be located in disordered parts.

Remarkably, we show here that TSA is part of a more extensive helicase structure. Indeed, the closest homologue to TSA is domain 2 of canonical helicases. TSA is known to reside in a larger ssDNA-binding domain, the boundaries of which have recently been defined, extending from residues 381 and 858 (Cheng *et al*., 2011). The structure of the N-terminal part of this domain from residue 381 to 569 has been solved and shown to be similar to subdomain 1A of SF1B helicases (Wright *et al*., 2012). Thus, the ssDNA-binding region of TraI contains three of the four canonical SF1B family subdomains, 1A, 2A and 2B. The parts of this domain, which have now been structurally characterized, constitute 70% of the entire sequence of the domain. Thus, it can be safely concluded that the ssDNA-binding domain is a vestigial helicase structure, which has lost its helicase activity but retained its ssDNA-binding capability.

Finally, the TSA structure demonstrates that translocation signals can be part of defined, well-characterized and larger structures supporting completely different function. This is also an unprecedented observation. Translocation signals are usually self-contained functional entity with no associated function other than mediating specific substrate recruitment to cognate transporters. Here we demonstrate that TSA is part of a vestigial helicase structure. Interestingly, TSB is also part of a helicase domain, but this time, the domain has retained helicase activity. These structural and functional features are clearly conserved among relaxases. Whether they will turn out to be found in other proteins remains to be demonstrated; however, TSA provides a template that might well prove of general use by transporters and secretion systems for interactions with their cognate substrates.

## Experimental procedures

### Strains, plasmids and primers

All *E. coli* strains used in this study are described in Table S2. Plasmids and Primers are described in Tables S3 and S4 respectively.

### Antibiotics, enzymes and reagents

Antibiotics were added at the indicated concentrations: ampicillin (100 μg ml^−1^), chloramphenicol (10 μg ml^−1^), kanamycin (40 μg ml^−1^), spectinomycin (75 μg ml^−1^), tetracycline (8 μg ml^−1^).

### DNA preparation and PCR amplification

Plasmid DNA was purified from *E. coli* cells with the QIAprep Spin Miniprep Kit (Qiagen, Hilden, Germany). Restriction endonucleases and Antarctic phosphatase were purchased from New England Biolabs, Beverly, MA, USA). T4 DNA ligase was purchased from Fermentas GmBH (St. Leon-Rot, Germany). DNA fragments for cloning were amplified using Phusion High-Fidelity DNA Polymerase (Finnzymes Oy, Espoo, Finland). Enzymes were used according to manufacturers' recommendations.

### Construction of the TSA overexpression plasmid

Expression construct of TSA (pCDF_TSA) with an N-terminal 6xHis tag and enterokinase cleavage site was generated by inserting the PCR-amplified DNA from R1-16 between the HindIII–BamHI sites in the pCDF1b vector.

### Construction of Cre-fusion plasmids

CreTraIN992 was constructed by ligating the SalI fragment from CreTraI(309–992) with SalI restricted CreTraIΔC1227. The insert for CreTraIN992 F was amplified with primers TraI_SFW1 and TraI_SRev5 from pOX38, cut with KpnI and ligated with CFB B. Two-step PCR was used to generate CreTraIN992 derivatives named for the corresponding point-mutations in TSA. In the first step primer sets 1 (TraISeqFW2 + Forward-Primer with the desired mutation) and 2 (TraI_SRev5 + Reverse-Primer with the desired mutation) were used to amplify two fragments from R1-16. In the second step these two fragments were annealed and amplified with primer set 3 (TraISeqFW2 + TraI_SRev5). The fragments were cut with SalI and religated with CreTraIΔC1227.

### Construction of expression plasmids

pGZTraIN992 derivatives were constructed in two steps: a 1.5 kb EcoRI/SalI fragment encoding the first 507 amino acids of TraI was isolated from pCG02 and ligated in pGZ119EH (Lessl *et al*., 1992) to generate pGZpartTraI. In step two the *traI* coding region for amino acids 507–992 was reconstructed by ligating the SalI fragment of desired CreTraIN992 mutants in pGZpartTraI linearized with SalI.

### Protein purification

For overexpression of R1 TraI_530–816, a 1 l culture of *E. coli* BL21(DE3) star carrying pCDF_TSA was grown at 37°C in LB medium containing 75 μg ml^−1^ spectinomycin to an *A*_600_ of 0.6, when IPTG was added to 1 mM final concentration. After an additional overnight incubation at 16°C, the cells were harvested by centrifugation and the pellets frozen at −80°C. Cells were thawed overnight at 4°C, resuspended in 15 ml of buffer I (50 mM sodium phosphate, 250 mM sodium chloride, 10 mM Imidazole, 0.02% sodium azide, pH = 7.5), and lysed by two passages through Emulsiflex-05 (AVESTIN). The soluble fraction was obtained by ultracentrifugation at 21000 g for 1h. The supernatant was filtered through a 0.45 μm filter prior to loading to a HisTrap column (GE Healthcare) equilibrated in buffer I. After washing the column with buffer I and 10% of buffer II (buffer I containing 500 mM Imidazole), the protein was eluted by applying a 50 ml gradient to 60% buffer II. Fractions containing protein were adjusted with 3 M ammonium sulphate to 1 M final concentration, combined and loaded onto a HiTrap Phenyl HP column equilibrated with buffer III (50 mM sodium phosphate, 100 mM sodium chloride, 1 mM EDTA pH = 8.0, 1 M ammonium sulphate, pH = 7.5). After washing the column with three column volumes of 50% buffer IV (50 mM sodium phosphate, 100 mM sodium chloride, 1 mM EDTA pH = 8.0, pH = 7.5), TSAΔC and full-length TSA were eluted sequentially by applying a 50–100% buffer IV gradient. Fractions containing protein were combined and concentrated with Amicon filter devices (Milipore) and the protein was injected to a 120 ml Sephacryl 200 HR column (GE Healthcare), equilibrated with buffer V (10 mM Tris pH = 7.5, 150 mM magnesium chloride). The protein eluted as a single peak. Fractions containing protein were concentrated to 15 mg ml^−1^ and either immediately used for crystallization or adjusted with glycerol to 40% (v/v) and stored at −80°C. The apparent molecular mass of the full-length protein of 33.7 kDa was confirmed by denaturating polyacrylamide gel electrophoresis and Coomassie blue staining. The N-terminal five residues (IISEPD) of the TSAΔ_cryst_ were assessed by Edman degradation analysis (PNAC Facility, University of Cambridge). SeMet-containing TSAΔ_cryst_ was produced as recommended by Molecular Dimension, and was purified using a similar protocol as for the wild-type protein.

### TSAΔ_cryst_ crystallization

TSAΔ_cryst_ was crystallized using the hanging drop vapour diffusion method (McCoy *et al*., 2007) using a solution of 0.1 M HEPES 7.5, 0.2 M sodium sulphate, 25% PEG 3350 as reservoir at a temperature of 16°C. Crystals appear after 3 days. TSAΔ_cryst_ crystallized in space group P41212, with unit cell dimensions of a = b = 109.17 Å, c = 56.80 Å, and diffracted to a resolution of 1.85 Å.

### Structure determination

The SeMet-containing protein crystals of TSAΔ_cryst_ was used for phasing. The data sets of the SeMet-TSA and native-TSA were collected at beamline ID14-2 at the European Synchrotron Radiation Facility (ESRF). A data set was collected at the wavelength of 0.8726 Å, and processed using the XDS suite. The unmerged HKL file was converted into an mtz file using POINTLESS. REINDEX and SCALA (Winn *et al*., 2011) were used for scaling and separating the anomalous pairs. The unmerged intensities were then converted into merged amplitudes using C-TRUNCATE (Winn *et al*., 2011). Heavy atoms search and SAD phases calculations were carried out using PHASER (McCoy *et al*., 2007). Phases were further improved by solvent flattening using DM (Winn *et al*., 2011). The solvent flattened phases were used for model building using BUCCANEER (Winn *et al*., 2011). Building of the initial model was completed manually using COOT. Restrained refinement and one round of TLS refinement was performed using REFMAC (Winn *et al*., 2011). The model was manually inspected and corrected in COOT using 2Fo-Fc maps (Emsley and Cowtan, 2004). The final model was validated using PROCHECK (Winn *et al*., 2011).

### Western blot analysis

For each sample a total of 2 ml mid-log-phase cells was pelleted and resuspended in 80 mM Tris-HCl (pH = 6.8), 6% DTT, 6% SDS, 12% glycerol and 0.04% bromophenol blue. Samples were heated to 96°C for 10 min and centrifuged afterwards. The equivalent of 0.1 *A*_600_ units was loaded on a 12.5% SDS-PAGE and run for 90 min at 10 mA. Transfer was done overnight at 4°C at 90 mA onto a milllipore immobilon-P membrane. Membranes were dried for 2 h at RT and soaked briefly in 100% methanol before blocking overnight at 4°C in 1× TBS (20 mM Tris-HCl pH = 7.5, 150 mM NaCl) with 3% BSA (albumin V). After washing two times for 5 min with TBS, primary anti-Cre rabbit polyclonal antibody (Novagen, 69050-3) was used in TBS with 3% BSA (1:10 000 dilution) for 2 h at RT. After washing [2 × 10 min with TBS, 1 × 10 min with TBS + 0.1% Tween20 (TBST)] membrane was incubated for 1 h at RT with peroxidase-conjugated anti-rabbit IgG (Sigma, A0545). After 4 × 10 min washing with TBST, detection was done with an ECL-Kit (GE Healthcare).

### CRAfT (Cre recombinase assay for translocation)

The Cre fusion reporter assay was performed as described previously (Lang *et al*., 2010). *E. coli* MS411 carrying the plasmids of interest and recipient CSH26Cm::LTL were used. Donors were selected on plates containing appropriate antibiotics (see Table S2). Transconjugants and recombinants were identified by plating serial dilutions on plates containing kanamycin (40 μg ml^−1^) and X-Gal (100 μg ml^−1^) or chloramphenicol (10 μg ml^−1^) respectively. Conjugation and protein translocation frequencies are calculated as transconjugants or recombinants per donor respectively.

### Infection studies with the male-specific phage R17

Fresh phage lysate was prepared as described previously (Lang *et al*., 2011). Liquid infection assays as described in Lang *et al*. (2011) were modified for 24-well cell culture plates (Greiner Bio-one). Briefly, 900 μl LB medium containing 2 mM CaCl_2_ and the appropriate antibiotics were inoculated to *A*_600_ 0.02 with the desired strain. R17 phage lysate (100 μl) was added to give a multiplicity of infection of 10. Cultures were grown at 37°C with shaking and cell lysis was determined by measuring the *A*_600_ from 0 to 240 min post infection using a xMark™ Microplate Spectrophotometer (Bio-Rad).
