# Supplementary material for: Structure of a translocation signal domain mediating conjugative transfer by type IV secretion systems
Source: Mol Microbiol. 2013 Jun 14;89(2):324–33. doi: 10.1111/mmi.12275 (PMC3912908; doi:10.1111/mmi.12275)
Supplement: Supplementary file 1 [file mmi0089-0324-sd1.pdf]

Table S1: Data collection and refinement statiscs

**Protein**	**TraI – TSA**
Cell	109.17, 109.17, 56.80
α, β, γ	90 90 90
Space group	P 4_1 2_1 2
Wavelength	0.8726 Å
Resolution	48.82 (1.85)
I/σ	26.3 (3.9)
R merge	0.073 (0.84)
CC 1/2	1 (0.86)
Completeness	100 (100)
Unique reflections	29933
Multiplicity	17.2 (17.5)
Anomalous multiplicity	9.2 (9.1)
FOM	0.45
**Refinement**	
R_{cryst}	0.18
R_{free}	0.22
Rms bond length	0.022
Rms Bond angles	2.209
**Ramachandran Plot**	
Number of residues	214
Preferred regions	208
Allowed regions	6
Outliers	0

Values in parentheses are for the highest resolution shell.

Table S2: *E. coli* K12 strains used in this study

**Strain**	**Description[a] and Reference**
E. coli MS411	*ilvG rfb-50 thi* (M. Schembri; DTU, Denmark)
E. coli CSH26Cm::LTL	Tc^R, CSH26 *galK::cat::loxP-Tet-loxP* (Lang, 2010)
E. coli BL21(DE3) Star	F- ompT hsdSB (rB-mB-) gal dcm rne131 (DE3) (Life Technologies)

[a]antibiotic resistance: Tc, tetracycline.

Table S3: Plasmids and primers used in this study

**Plasmid**	**Description[a] and Reference**
Cre-fusion plasmids	
CFP B	Amp^R; *cre* from phage P1 cloned into the *Nhe*I and *Sal*I site of pBR322 (Parker and Meyer, 2007)
CreTraI(309-992)	Amp^R; CFP B with partial R1 *traI* encoding residue 309-992 (Lang 2010)
CreTraIDC1227	Amp^R; CFP B with partial R1 *traI* encoding for TraI missing the last 1227 residues (Lang 2010)
CreTraIN992	Amp^R; CFP B with partial R1 *traI* encoding residue 1-992 (this study)
CreTraIN992_A593V	Amp^R; CreTraIN992 derivative encoding a A593V mutation (this study)
CreTraIN992_H626L	Amp^R; CreTraIN992 derivative encoding a H626L mutation (this study)
CreTraIN992_D714A	Amp^R; CreTraIN992 derivative encoding a D714A mutation (this study)

CreTraIN992_D714N	Amp^R; CreTraIN992 derivative encoding a D714N mutation (this study)
CreTraIN992_R717Q	Amp^R; CreTraIN992 derivative encoding a R717Q mutation (this study)
CreTraIN992_Q736A	Amp^R; CreTraIN992 derivative encoding a Q736A mutation (this study)
CreTraIN992_Q736N	Amp^R; CreTraIN992 derivative encoding a Q736N mutation (this study)
CreTraIN992_S739A	Amp^R; CreTraIN992 derivative encoding a S739A mutation (this study)
CreTraIN992_S739N	Amp^R; CreTraIN992 derivative encoding a S739N mutation (this study)
CreTraIN992_V746A	Amp^R; CreTraIN992 derivative encoding a V746A mutation (this study)
CreTraIN992_V746N	Amp^R; CreTraIN992 derivative encoding a V746N mutation (this study)
CreTraIN992_S757T	Amp^R; CreTraIN992 derivative encoding a S757T mutation (this study)
CreTraIN992 F	Amp^R; CFP B with partial F *traI* encoding residue 1-992 (this study)
Expression Plasmids	
pCG02	Cm^R; pGZ119EH with partial R1 *traI* encoding residue 1-992 (Lang, 2010)
pGZTraIN992_A593V	Cm^R; pGZ119EH with partial R1 *traI* encoding residue 1-992 and a A593V mutation (this study)
pGZTraIN992_H626L	Cm^R; pGZ119EH with partial R1 *traI* encoding residue 1-992 and a H626L mutation (this study)
pGZTraIN992_D714 N	Cm^R; pGZ119EH with partial R1 *traI* encoding residue 1-992 and a D714N mutation (this study)
pGZTraIN992_Q736 N	Cm^R; pGZ119EH with partial R1 *traI* encoding residue 1-992 and a Q736N mutation (this study)
pGZTraIN992_S739N	Cm^R; pGZ119EH with partial R1 *traI* encoding residue 1-992 and a S739N mutation (this study)
Overexpression Plasmids	
pCDF_TSA	Spect^R; pCDF1b with partial R1 traI encoding residue 530-816 (this study)
Conjugative Plasmids	
R1-16	Km^R; IncFII, *fin-* (Goebel *et al.*, 1977)
R1-16D*traI*	Km^R, Tc^R; IncFII, *traI::tetRA* (Lang, 2010)
pOX38	Km^R, IncFI, derivative of F (Chandler & Galas, 1983)

[a]antibiotic resistance: Amp, ampicillin; Cm, chloramphenicol; Km, kanamycin; Spect, spectinomycine; Tc, tetracycline.

Table S4: Primers used in this study

Primer	Primer sequence 5´-3´
TraI_SFW1	CATGTA*GGTACC*AGTATCGCGCAGGTCAGA
TraI_SRev5	CATATT*GGTACC*TTACCCCTGTACCACCGTGAAAC
TraISeqFW2	ACCGTCGCTCGCAGAT
TraI530_FW	AGTC*GGATCC*CGTACAGGTCCTGATAACC
TraI816_Rev	AGTC*AAGCTT*CTAGGAATACAGCCGGACATC
Forward Primer with desired mutation	
TraI_V593FW	GCCAGCGTGAAAG**T**CGGAGAAGAGAGC
TraI_L626FW	CGTGCTCGGAC**T**CCCTGAGGTGAC
TraI_A714FW	TGCCGGTGGCAG**C**CGGCGAGCGACTG
TraI_N714FW	TGCCGGTGGCA**A**ACGGCGAGCGACTG
TraI_Q717FW	GCAGACGGCGAGC**A**ACTGAGGGTGACAG
TraI_A736FW	GGTGACCGCCTG**GCG**GTGGCATCCGTCAGT
TraI_N736FW	GGTGACCGCCTG**A**A**C**GTGGCATCCGTCAGT
TraI_A739FW	CTGCAGGTGGCA**G**CCGTCAGTGAAGATG
TraI_N739FW	CTGCAGGTGGCA**AA**CGTCAGTGAAGATG
TraI_A746FW	AGATGCGATG**G**CGGTTGTTGTGCC
TraI_N746FW	AGATGCGATGA**AT**GTTGTTGTGCC
TraI_T757FW	GGGCGGGCTGAACCGGCC**A**CCCTGCCTGTGAGCGAT

Reverse Primer with desired mutation	
TraI_V593Rev	GCTCTCTTCTCCG**A**CTTTCACGCTGGC
TraI_L626Rev	GTCACCTCAGGG**A**GTCCGAGCACG
TraI_A714Rev	CAGTCGCTCGCCG**G**CTGCCACCGGCA
TraI_N714Rev	CAGTCGCTCGCCGT**T**TGCCACCGGCA
TraI_Q717Rev	CTGTCACCCTCAGT**T**GCTCGCCGTCTGC
TraI_A736Rev	ACTGACGGATGCCAC**CGC**CAGGCGGTCACC
TraI_N736Rev	ACTGACGGATGCCAC**G**T**T**CAGGCGGTCACC
TraI_A739Rev	CATCTTCACTGACGG**C**TGCCACCTGCAG
TraI_N739Rev	CATCTTCACTGACG**TT**TGCCACCTGCAG
TraI_A746Rev	GGCACAACAACCG**C**CATCGCATCT
TraI_N746Rev	GGCACAACAAC**AT**TCATCGCATCT
TraI_T757Rev	ATCGCTCACAGGCAGGG**T**GGCCGGTTCAGCCCGCCC

[a]italics: enzyme restriction site; bold: desired mutation

Accession numbers: F *traI* (AP001918), R1 *traI* (AY423546)

Supplementary Figures

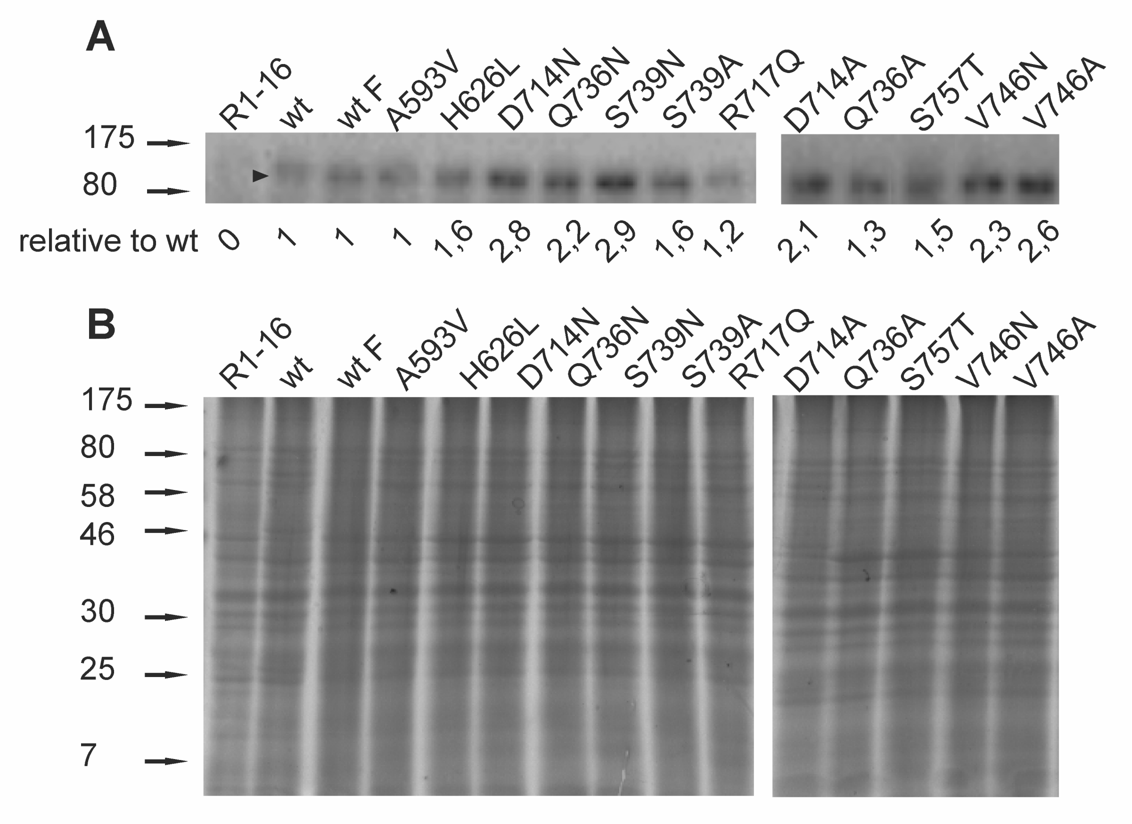

Fig. S1: Western gel analysis of Cre fusion proteins. The equivalent of 0,1 A600 units was applied to each well, resolved with 12.5% SDS-PAGE, and transferred to membranes as described in Experimental procedures. Proteins detected with rabbit anti-Cre antibody and peroxidase-conjugated anti-rabbit antibody are shown in (A). Relative protein abundance compared to wild type Cre-TraIN992 is indicated below. To compare the abundance of total protein loaded in each lane, the equivalent of 0,1 A600 units was subjected to SDS-PAGE analysis and visualized by Coomassie staining (B).

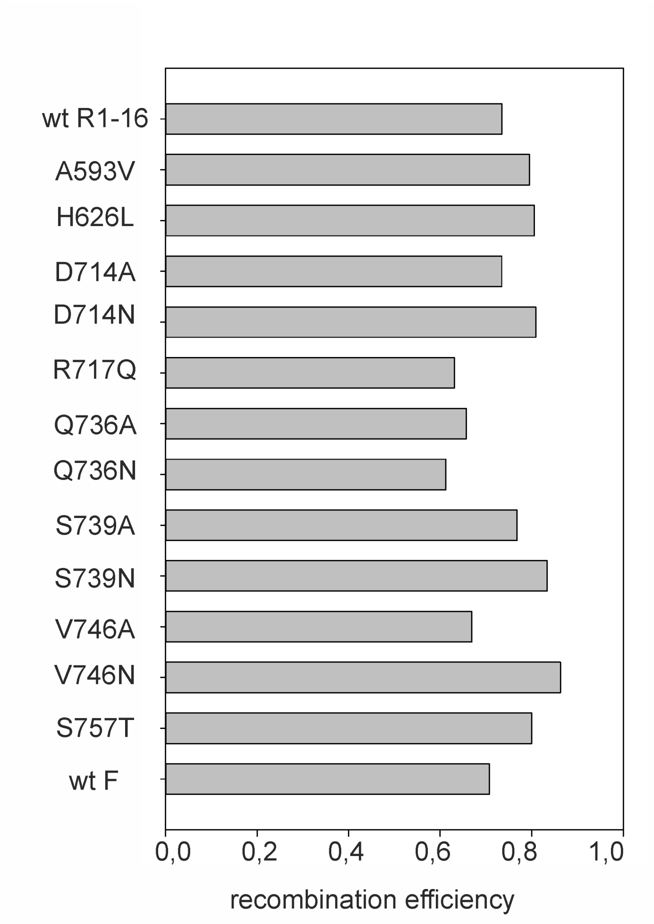

Fig. S2: Cre-TraIN992 and mutants thereof catalyze recombination after transformation of the CRAfT indicator strain with each expression plasmid. Approximately 50ng DNA of each construct (*right*) was introduced into the indicator strain. Selection for transformed cells was performed on 100 µg/ml Amp LB agar plates and recombinants were detected with 10µg/ml Cm . Recombination efficiency was calculated as the number of CmR colonies divided by the number of AmpR colonies.

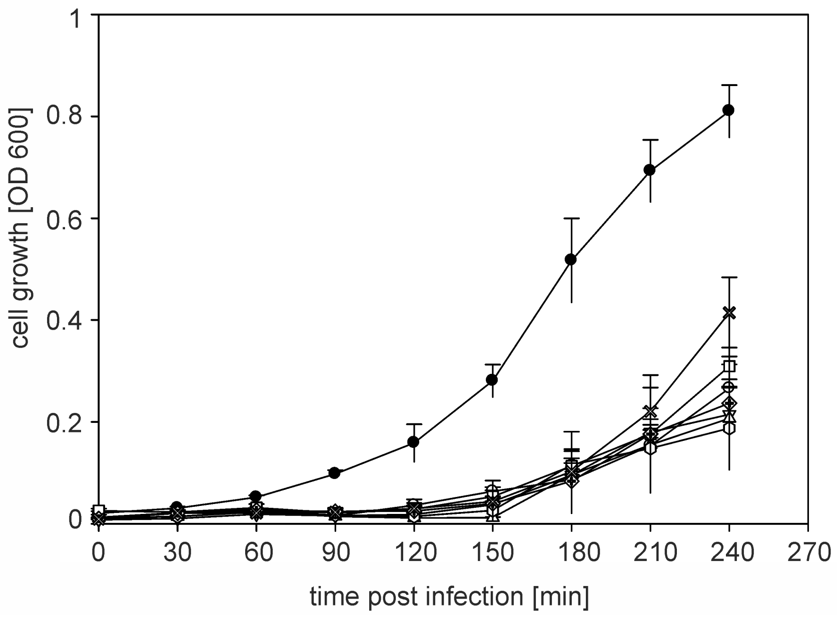

Fig. S3: The N-terminal activation domain TraI N_{1-992} (▽) and variants thereof are sufficient to complement R1-16D*traI* (●) carrying MS411 host cells for R17 infection to levels equal to wild type MS411 [R1-16] (○). Phage infection was performed as described in Experimental procedures. Variants are indicated with the following symbols: A593V (✕), H626L (△), D714N (⬡), Q736N (◇), S739N (□)
